# The Mediating Role of Sleep Quality in Associations of Smartphone Addiction and Time-Specific Use With Prenatal Depression and Anxiety: Cross-Sectional Observational Study

**DOI:** 10.2196/100064

**Published:** 2026-10-30

**Authors:** Xiangxin Zhang, Mixia Ouyang, Xiaoli Wu, Xinghua Yang, Xinyi Wang, Yuxinqiao Cao, Mingmin Xu, Yanling Shu, Mingyang Wu

**Affiliations:** 1 Department of Maternal and Child Health, Xiangya School of Public Health, Central South University, No. 172 Tongzipo Road, Changsha, Hunan, 410013, China, 86 1-597-147-1776; 2 Hunan Provincial Maternal and Child Health Hospital, Changsha, Hunan, China; 3 Department of Maternal and Child Health, School of Public Health, Tongji Medical College, Huazhong University of Science and Technology, Wuhan, Hubei, 430030, China

**Keywords:** smartphone addiction, smartphone use, sleep quality, antenatal anxiety, antenatal depression, prenatal depression, prenatal anxiety, time-specific smartphone use, restricted cubic spline, mediating effect

## Abstract

**Background:**

Prenatal depression and anxiety have emerged as pressing global public health challenges. In the digital era, excessive smartphone use may exert complex effects on maternal mental health. Prior research has predominantly focused on adolescents, whereas evidence among pregnant women, particularly regarding total weekly use and use during critical circadian windows, such as after waking and before bedtime, remains limited. Furthermore, the mediating role of sleep quality in these associations has yet to be systematically examined.

**Objective:**

This study aimed to investigate the associations of smartphone addiction and time-specific smartphone use with prenatal depression and anxiety, and to assess the mediating role of sleep quality in these associations.

**Methods:**

This cross-sectional observational study enrolled 537 pregnant women. Smartphone use characteristics, sleep quality, and prenatal depression and anxiety were assessed using the Smartphone Addiction Scale-Short Version (SAS-SV), objective total weekly smartphone use recorded by the device, and self-reported duration of smartphone use after waking and duration of bedtime smartphone use; the Pittsburgh Sleep Quality Index (PSQI), the Patient Health Questionnaire-9 (PHQ-9), and the Generalized Anxiety Disorder-7 (GAD-7), respectively. Binary logistic regression was used to estimate associations, restricted cubic spline (RCS) models were used to explore nonlinear patterns, and exploratory mediation analyses were used to examine whether sleep quality statistically accounted for part of the observed associations.

**Results:**

The prevalence of moderate-to-severe prenatal depression and anxiety was 8.8% (47/537) and 5.8% (31/537), respectively. After adjusting for covariates, smartphone addiction was associated with higher odds of depression (odds ratio [OR] 3.461, 95% CI 1.771‐7.100) and anxiety (OR 7.738, 95% CI 3.064‐23.172). Duration of smartphone use after waking ≥61 minutes and duration of bedtime smartphone use ≥91 minutes were also associated with higher odds of affective disorders. RCS analyses further revealed distinct nonlinear patterns across different exposure dimensions: duration of smartphone use after waking displayed U-shaped and J-shaped associations with depression and anxiety, respectively, while total weekly use displayed a U-shaped association with anxiety. Exploratory mediation analyses showed that the indirect pathways through sleep quality were statistically significant for the associations of bedtime smartphone use, weekly smartphone use, and smartphone addiction with prenatal depression, with mediation proportions of 44.89% (0.0065/0.0145), 31.30% (0.0056/0.0183), and 31.48% (0.0415/0.1315), respectively. The corresponding proportions in the anxiety models were 35.83% (0.0044/0.0123), 27.05% (0.0039/0.0144), and 22.73% (0.0271/0.1187).

**Conclusions:**

Smartphone addiction and excessive smartphone use after waking and before bedtime were associated with higher odds of prenatal depression and anxiety. Sleep quality may statistically account for part of these associations, but longitudinal studies are needed to establish temporal ordering and causal pathways.

## Introduction

Pregnancy represents a critically vulnerable period in a woman’s life course, both physiologically and psychologically [1-3]. During this period, women not only endure physiological challenges such as dramatic hormonal fluctuations and early pregnancy symptoms, but also face multiple psychosocial stressors, including shifts in social roles, restructuring of family relationships, and concerns about fetal well-being [4,5]. Although pregnancy is generally regarded as a positive life event, it is frequently accompanied by significant affective disturbances. A systematic review reported that the global average prevalence of prenatal depression is approximately 28.5%, which is substantially higher than that of postpartum depression [6]. In mainland China, a systematic review encompassing 271 studies and 369,477 women found that the average prevalence of prenatal anxiety reached 17.4% [7]. These common prenatal affective disorders, if left unaddressed, can substantially increase the risk of adverse obstetric outcomes, such as preterm birth, low birth weight, and preeclampsia [8,9].

In the digital era, smartphones have become an integral extension of daily life. For pregnant women, smartphones serve as important tools for accessing health information and maintaining social support. Healthy smartphone use can, to some extent, alleviate the sense of isolation and uncertainty brought about by the temporary disruption of social roles [10]. Conversely, the fragmented entertainment offered by smartphones can easily trigger overuse, blurring the self-perceived boundary between functional use and pathological dependence [11]. Against this backdrop, the positive mental health effects of smartphone use may be reversed, turning it into a potential risk factor that jeopardizes maternal prenatal mental health [12].

Smartphone addiction, also referred to as problematic smartphone use, is defined as excessive dependence on smartphones, characterized by core features of behavioral addiction such as withdrawal anxiety, increased tolerance, and impaired social functioning [13,14]. In recent years, the adverse effects of smartphone addiction on mental health have been widely documented across diverse populations. Large-scale studies among adolescents, in particular, have demonstrated significant positive associations of problematic smartphone use with depressive and anxiety symptoms as well as sleep disturbance [15-17]. Moreover, several longitudinal studies have shown that baseline levels of smartphone addiction prospectively predict the worsening of anxiety symptoms and the continued deterioration of sleep quality during follow-up, suggesting that smartphone addiction may not merely be a consequence of psychological distress but a potential cause that precipitates or exacerbates affective disorders [18,19].

Although previous research has established negative associations between smartphone addiction and mental health in adolescents [20,21], studies examining the association between smartphone addiction and prenatal affective disorders among pregnant women, a population characterized by marked physiological and psychological fluctuations, remain scarce. In addition, few studies have distinguished total smartphone use time from use during specific periods. This distinction may be theoretically relevant: circadian rhythm disruption plays a key role in the onset, recurrence, and treatment response of mood disorders, and the central role of circadian rhythms in emotional regulation is widely recognized [22,23]. Screen exposure during different circadian phases may influence emotional states through distinct neurobiological pathways. Specifically, bedtime screen exposure is closely associated with reduced sleep quality [24], whereas screen exposure after waking may have different effects on the daytime stress regulation system [25]. Compared with absolute usage duration, whether screen exposure during key circadian windows, such as bedtime and after waking, is associated with prenatal depressive and anxiety symptoms has yet to be systematically investigated.

In recent years, studies on adolescents have shown that smartphone use is associated with reduced sleep quality and emotional problems [20,26,27]. Experimental studies have further found that nighttime blue light exposure can prolong sleep latency and cause a circadian phase shift by suppressing melatonin secretion [28], while the cognitive and emotional arousal triggered by immersive screen exposure may also interfere with sleep onset and maintenance [29]. It is worth noting that sleep quality in pregnant women undergoes a physiological deterioration as pregnancy progresses, with the prevalence of sleep disturbances rising significantly from the first trimester to the third trimester [30]. This vulnerability may stem from the combined effects of hormonal fluctuations, increased nocturia, and physical discomfort [31]. Furthermore, poor sleep quality is associated with an increased risk of prenatal depression and anxiety [32,33], and its neural mechanisms may involve the effects of sleep deprivation on prefrontal cortex function and amygdala reactivity [34,35]. Based on this prior evidence and biological plausibility, it is reasonable to infer that sleep quality may mediate the relationship between smartphone use and prenatal affective disorders. However, current research on this topic remains scarce, particularly studies that comprehensively assess multiple dimensions, such as smartphone use at specific time points and smartphone addiction.

To address this research gap, this study aims to investigate the associations between smartphone addiction and usage during specific time periods and prenatal depression and anxiety, and to explore the mediating effect of sleep quality on these associations, to provide evidence-based support for the development of digital health guidelines and clinical intervention strategies during pregnancy.

## Methods

### Study Population

This study used a cross-sectional design and was conducted in the obstetrics outpatient clinic and inpatient ward of Hunan Provincial Maternal and Child Health Hospital. Participants were recruited using convenience sampling. The survey was administered by uniformly trained interviewers, who explained the study’s purpose and procedures to eligible pregnant women while they were waiting to give birth in the admission area or in the ward. After obtaining informed consent, the interviewers provided one-on-one guidance to help participants scan a QR code to complete the electronic questionnaire, which was filled out and submitted on the spot. Investigators provided necessary explanations and assistance but did not interfere with the respondents’ answers. Inclusion criteria were as follows: (1) permanent residents of Changsha, (2) all prenatal checkups completed at the study hospital, (3) age ≥18 years, (4) singleton pregnancy. Exclusion criteria were (1) severe cognitive impairment or mental illness preventing effective communication or understanding of the questionnaire content, and (2) presence of legally reportable infectious diseases such as HIV, syphilis, or hepatitis B. Participants who met all inclusion criteria and none of the exclusion criteria were formally enrolled in the study after signing the informed consent form.

All data in this study were collected by having participants scan a QR code to complete a structured questionnaire. All key research variables in the questionnaire were marked as required fields; participants could not submit the questionnaire unless they had completed all required items. Consequently, none of the variables analyzed in this study had missing values, and all 537 cases ultimately included in the analysis were valid questionnaires.

### Ethical Considerations

The study protocol, encompassing participant recruitment procedures, informed consent procedures, data collection, and quality control methods, was reviewed and approved by the Ethics Committee of the Xiangya School of Public Health, Central South University (approval number: XYGW-2024‐19) and the Ethics Committee of the Hunan Provincial Maternal and Child Health Care Hospital (approval number: 2023-S189). After fully understanding the study’s objectives, procedures, potential risks, and benefits, all participants voluntarily signed a paper informed consent form and were informed of their right to withdraw from the study at any time without condition. No remuneration or compensation was provided to any participant in this study.

### Exposure Measurement

Smartphone addiction tendency was assessed using the Smartphone Addiction Scale-Short Version (SAS-SV). The scale consists of 10 items rated on a 6-point Likert scale (1=“strongly disagree” to 6=“strongly agree”), yielding a total score ranging from 10 to 60, with higher scores indicating a greater risk of smartphone addiction. According to the developers’ recommendation, a cut-off score of 33 was adopted to define a high risk of addiction for females. The SAS-SV has demonstrated good psychometric properties, with an internal consistency Cronbach α coefficient of 0.91 [36]. The scale has been widely applied and validated in Chinese populations and is suitable for the rapid screening of smartphone dependence among adults [37].

Time-specific smartphone use was assessed using self-developed items, including duration of bedtime smartphone use and duration of smartphone use after waking. Bedtime smartphone use was defined as the cumulative duration of smartphone use from the moment participants lay down in bed with the intention to sleep until they actually fell asleep. Smartphone use after waking was defined as the cumulative duration of use from the moment participants awoke in the morning until they got out of bed. Both definitions were clearly stated in the questionnaire and were verbally explained in a standardized manner by trained investigators before questionnaire completion. Investigators specifically emphasized that the reported duration should only include time spent actively using the phone while lying in bed, rather than the total time spent in bed including nonuse states. Meanwhile, participants were instructed to recall their typical usage patterns over the past week rather than relying on a single day’s experience. Both measures were specific to nighttime sleep periods and did not include daytime naps, and were recorded in minutes. Weekly smartphone use was obtained by asking participants to check the average daily usage duration of all applications over the past week through the screen time management feature built into their smartphone system settings (corresponding to “Screen Time” or “Healthy Phone Usage Time” or “App Management Time” in Android and Apple iOS systems, respectively), and was recorded in hours. According to the current distribution of data and previous studies on smartphone use duration, we classified participants into 4 consecutive groups as follows: (1) duration at bedtime [25]: 0‐30 minutes, 31‐60 minutes, 61‐90 minutes, and ≥91 minutes; (2) duration after waking [38]: 0‐1 minutes, 2‐10 minutes, 11‐60 minutes, and ≥61 minutes; (3) duration of total week [39,40]: 0‐14 hours/week, 15‐28 hours/week, 29‐49 hours/week, and ≥50 hours/week.

Participants’ sleep quality over the past month was assessed using the Pittsburgh Sleep Quality Index (PSQI). Developed by Buysse et al [41], the PSQI comprises 7 dimensions: subjective sleep quality, sleep latency, sleep duration, habitual sleep efficiency, sleep disturbances, use of sleep medication, and daytime dysfunction. The total score, ranging from 0 to 21, is calculated as the sum of all component scores. Based on the threshold established by previous studies, a total score of ≥7 was defined as poor sleep quality in this study [42,43].

### Outcome Measurement

Depressive symptoms over the past 2 weeks were assessed using the Patient Health Questionnaire-9 (PHQ-9). Developed by Kroenke et al [44] based on the DSM-IV diagnostic criteria for depression, the PHQ-9 comprises nine items. Each item asks respondents to rate the frequency of each symptom over the past 2 weeks on a 4-point Likert scale (0=“not at all” to 3=“nearly every day”), yielding a total score ranging from 0 to 27, with higher scores indicating more severe depressive symptoms. The PHQ-9 has demonstrated good reliability and validity among Chinese pregnant women [45]. As prior research has shown that moderate-to-severe depressive symptoms may lead to more adverse outcomes, a cut-off score of ≥10 was adopted in this study to define moderate-to-severe depressive symptoms, in line with previous studies [46,47]. Hereafter, the term “prenatal depression” refers to moderate-to-severe prenatal depressive symptoms.

Anxiety symptoms were measured using the Generalized Anxiety Disorder-7 (GAD-7). Developed by Spitzer et al [48], the GAD-7 is designed for rapid screening of generalized anxiety symptoms over the past 2 weeks. The scale consists of 7 self-reported items, each requiring respondents to rate the frequency of anxiety symptoms over the past 2 weeks on a 4-point Likert scale (0=“not at all” to 3=“nearly every day”), with total scores ranging from 0 to 21, higher scores reflecting more severe anxiety symptoms. Given that moderate-to-severe anxiety symptoms may be associated with more detrimental effects, a cut-off score of ≥10 was used to define moderate-to-severe anxiety, consistent with previous research [49,50]. Hereafter, the term “prenatal anxiety” refers to moderate-to-severe prenatal anxiety symptoms.

### Covariates

Based on previous studies [12,51-55], the following variables that may influence smartphone use and prenatal mental health outcomes were included as covariates in the analyses, covering 3 domains: sociodemographic characteristics, behavioral and lifestyle factors, and health and pregnancy-related characteristics. Sociodemographic characteristics included maternal age (years), maternal educational attainment (junior high school or below, high school or vocational high school, and college or above), and paternal educational attainment (junior high school or below, high school or vocational high school, and college or above). Behavioral and lifestyle factors included prepregnancy smoking status within the 6 months before pregnancy (yes or no), prepregnancy alcohol consumption within the 6 months before pregnancy (yes or no), frequency of physical exercise during pregnancy (<once per week, 1‐3 times per week, 4‐6 times per week, and ≥ once per day), and frequency of social media posting on platforms such as WeChat (Tencent) and Xiaohongshu (Xingyin Information Technology Ltd.) during pregnancy (less than once every 6 months, 2‐5 times per year, 1‐2 times per month, 3‐4 times per month, and several times per week). Health- and pregnancy-related characteristics included gestational age (weeks), prepregnancy BMI (kg/m²), gestational weight gain (kg), gestational diabetes mellitus (yes or no), abortus (times), and gravida (times).

### Statistical Analysis

Descriptive statistics were computed for the dataset, with continuous variables presented as means and SD and categorical variables expressed as counts and percentages. Between-group comparisons were performed using independent-samples *t* tests for continuous variables and chi-square tests for categorical variables. Pearson correlation coefficients were used to assess the bivariate associations among total weekly smartphone use duration, duration of smartphone use after waking, duration of bedtime smartphone use, SAS-SV score, PHQ-9 score, and GAD-7 score.

Subsequently, binary logistic regression models were fitted separately for each smartphone-use dimension and outcome. Two models were constructed to verify the robustness of the associations: Model 1 was a crude model that assessed the initial associations without adjusting for any covariates; Model 2 was a fully adjusted model that included maternal age, maternal educational attainment, paternal educational attainment, prepregnancy BMI, gestational weight gain, gestational age, gestational diabetes mellitus, gravida, abortus, prepregnancy smoking status, prepregnancy alcohol consumption, frequency of physical exercise during pregnancy, and frequency of social media posting during pregnancy as covariates. Results were expressed as odds ratios (ORs) with their 95% CIs.

To further explore potential nonlinear dose-response relationships between smartphone use, sleep quality, and prenatal affective disorders, restricted cubic spline (RCS) functions were fitted on the basis of the above logistic regression models. Following previous studies, 4 knots were placed at the fifth, 35th, 65th, and 95th percentiles, and the median of each independent variable was set as the reference value [56-58]. Finally, mediation models were established to exploratorily examine the indirect pathways of sleep quality in the associations between multidimensional smartphone use and prenatal affective disorders. In our mediation analysis, we clearly distinguished between the total effect, the direct effect, and the indirect effect based on the effect decomposition framework. We used the continuous scores of the PHQ-9 and GAD-7 as outcome variables to preserve the informational integrity of the original scales and enhance the statistical power for detecting indirect effects. Point estimates for direct and indirect effects were obtained using linear regression models, and their 95% CIs were estimated via 1000 Monte Carlo simulations; the mediation proportion was calculated by dividing the indirect effect by the total effect. All models were adjusted for all abovementioned covariates. In addition, to examine the impact of outcome definition on the stability of mediation results, we conducted a sensitivity analysis based on a binary outcome (PHQ-9 and GAD-7 score ≥10). Given the cross-sectional design of this study, the results of the mediation analysis should be interpreted as an exploratory decomposition of association pathways rather than evidence of causal mechanisms.

Given the limited number of events for moderate-to-severe depression (n=47) and anxiety (n=31), two sensitivity analyses were conducted to assess the robustness of the main findings. First, Firth penalized maximum likelihood estimation was applied to refit all models to reduce small-sample bias in rare-events logistic regression [59]. Second, the threshold for the PHQ-9 and GAD-7 outcomes was lowered from ≥10 to ≥5 (ie, including those with mild and above depression and anxiety symptoms) to evaluate whether the observed associations were sensitive to the choice of the cutoff for symptom severity.

Data analyses were performed using SPSS 26.0 (IBM Corp) and R 4.4.1 (R Foundation for Statistical Computing). The primary R packages used included *dplyr*, *rms*, *mediation*, and *ggplot2*. The significance level was set at α=.05, and *P*<.05 was considered statistically significant.

## Results

### Baseline Characteristics of Participants

Table 1 presents the baseline characteristics of the study participants. A total of 537 pregnant women were included, with a mean age of 31.7 (4.0) years. Moderate-to-severe prenatal depression and anxiety were present in 47 (8.8%) and 31 (5.8%) participants, respectively. The mean SAS-SV score (SD) in the moderate-to-severe depression group was 36.4 (9.4), significantly higher than that in the nonmoderate-to-severe depression group (30.8, SD 8.9). The moderate-to-severe depression group also had longer weekly screen time and significantly longer durations of smartphone use after waking and before bedtime compared with the nonmoderate-to-severe depression group (*P*=.02 and *P*=.002, respectively). In addition, the PSQI score in the moderate-to-severe depression group was significantly higher than that in the nonmoderate-to-severe depression group (*P*<.001). The pattern of differences between the moderate-to-severe anxiety group and the nonmoderate-to-severe anxiety group was generally similar. The overall detection rate of sleep disturbance was 46.2% (n=248), and the detection rates in both the moderate-to-severe depression group and the moderate-to-severe anxiety group were significantly higher than those in their respective control groups (*P*=.003 and *P*=.008, respectively).

**Table 1.** Baseline characteristics of the study participants (N=537)^a^.

Variables	Total	Prenatal depression (PHQ-9^b^ score≥10)			Prenatal anxiety (GAD-7^c^ score≥10)		
		No	Yes	*P* value	No	Yes	*P* value
Age, mean (SD) years	31.7 (4.0)	31.7 (4.0)	32.0 (3.6)	.60	31.7 (4.0)	32.3 (4.5)	.40
BMI, mean (SD) kg/m²	21.0 (2.9)	21.1 (2.9)	20.8 (3.0)	.48	21.0 (2.9)	21.2(2.8)	.78
Gravida, mean (SD) times	2.0 (1.1)	2.0 (1.1)	2.1 (1.4)	.29	2.0 (1.1)	2.0 (1.2)	.85
Abortus, mean (SD) times	1.6 (0.9)	1.5 (0.8)	1.7 (1.0)	.30	1.6 (0.9)	1.7 (0.8)	.55
Weight gain during pregnancy, mean (SD) kg	15.2 (5.9)	15.3 (5.9)	14.0 (5.4)	.13	15.2 (5.8)	14.6 (7.0)	.56
Gestational age, mean (SD) weeks	38.87 (1.43)	38.86 (1.44)	38.98 (1.39)	.86	38.87 (1.43)	38.91 (1.46)	.94
Gestational diabetes mellitus, n (%)				.58			.46
No	441 (82.1)	401 (81.8)	40 (85.1)		414 (81.8)	27 (87.1)	
Yes	96 (17.9)	89 (18.2)	7 (14.9)		92 (18.2)	4 (12.9)	
Maternal education attainment, n (%)				**.**02^d^			.61
Junior high school or below	19 (3.5)	15 (3.1)	4 (8.5)		17 (3.4)	2 (6.5)	
High school or vocational high school	43 (8.0)	36 (7.3)	7 (14.9)		40 (7.9)	3 (9.7)	
College or above	475 (88.5)	439 (89.6)	36 (76.6)		449 (88.7)	26 (83.9)	
Paternal education attainment, n (%)				.57			.29
Junior high school or below	30 (5.6)	26 (5.3)	4 (8.5)		29 (5.7)	1 (3.2)	
High school or vocational high school	58 (10.8)	52 (10.6)	6 (12.8)		52 (10.3)	6 (19.4)	
College or above	449 (83.6)	412 (84.1)	37 (78.7)		425 (84.0)	24 (77.4)	
Prepregnancy smoking, n (%)				>.99			.09
No	511 (95.2)	466 (95.1)	45 (95.7)		484 (95.7)	27 (87.1)	
Yes	26 (4.8)	24 (4.9)	2 (4.3)		22 (4.3)	4 (12.9)	
Prepregnancy alcohol consumption, n (%)				.48			.41
No	373 (69.5)	343 (70.0)	30 (63.8)		354 (70.0)	19 (61.3)	
Yes	164 (30.5)	147 (30.0)	17 (36.2)		152 (30.0)	12 (38.7)	
Frequency of physical exercise, n (%)				.25			.30
Less than once a week	223 (41.5)	197 (40.2)	26 (55.3)		206 (40.7)	17 (54.8)	
1‐3 times a week	199 (37.1)	186 (38.0)	13 (27.7)		192 (37.9)	7 (22.6)	
4‐6 times a week	62 (11.5)	58 (11.8)	4 (8.5)		59 (11.7)	3 (9.7)	
Once a day or more	53 (9.9)	49 (10.0)	4 (8.5)		49 (9.7)	4 (12.9)	
Frequency of social media posts, n (%)				.07			.33
Less than once every six months	171 (31.8)	159 (32.4)	12 (25.5)		161 (31.8)	10 (32.3)	
2‐5 times a year	144 (26.8)	125 (25.5)	19 (40.4)		132 (26.1)	12 (38.7)	
1‐2 times a month	147 (27.4)	138 (28.2)	9 (19.1)		143 (28.3)	4 (12.9)	
3‐4 times a month	53 (9.9)	50 (10.2)	3 (6.4)		49 (9.7)	4 (12.9)	
Several times a week	22 (4.1)	18 (3.7)	4 (8.5)		21 (4.2)	1 (3.2)	
PSQI^e^ total score, mean (SD) points	7.6 (3.3)	7.3 (3.2)	9.9 (3.6)	<.001	7.4 (3.3)	9.9 (3.4)	<**.**001
Poor sleep quality, n (%)				.003			.008
No	289 (53.8)	274 (55.9)	15 (31.9)		280 (55.3)	9 (29.0)	
Yes	248 (46.2)	216 (44.1)	32 (68.1)		226 (44.7)	22 (71.0)	
Weekly smartphone use, mean (SD) hours	31.3 (27.6)	30.8 (26.8)	36.4 (34.2)	.18	30.8 (26.8)	39.0 (37.4)	.11
SAS-SV^f^ total score, mean (SD) points	31.3 (9.1)	30.8 (8.9)	36.4 (9.4)	<.001	30.8 (9.0)	38.3 (7.6)	<.001
Smartphone addiction, n (%)				<.001			<.001
No	309 (57.5)	295 (60.2)	14 (29.8)		303 (59.9)	6 (19.4)	
Yes	228 (42.5)	195 (39.8)	33 (70.2)		203 (40.1)	25 (80.6)	
Smartphone use after waking, mean (SD) minutes	29.1 (50.6)	27.4 (50.9)	46.3 (44.2)	.02	28.1 (51.0)	45.0 (40.3)	.07
Bedtime smartphone use, mean (SD) minutes	73.1 (53.4)	70.9 (52.1)	96.5(61.6)	.002	71.3 (52.4)	102.9 (62.4)	.001

a Continuous variables (age, BMI, gravida, abortus, weight gain during pregnancy, gestational age, PSQI total score, weekly smartphone use, SAS-SV total score, smartphone use after waking, and bedtime smartphone use) were analyzed using an independent samples *t* test, while categorical variables (gestational diabetes mellitus, maternal education attainment, paternal education attainment, prepregnancy smoking, prepregnancy alcohol consumption, frequency of physical exercise, frequency of social media posts, poor sleep quality, and smartphone addiction) were analyzed using the chi-square test.

b PHQ-9: Patient Health Questionnaire-9.

c GAD-7: Generalized Anxiety Disorder-7.

d Statistical significance (*P*<.05).

e PSQI: Pittsburgh Sleep Quality Index.

f SAS-SV: Smartphone Addiction Scale-Short Version.

### The Correlation between Different Dimensions of Smartphone Use and Prenatal Affective Disorders

To preliminarily explore the bivariate associations between multidimensional smartphone use characteristics during pregnancy (SAS-SV total score, weekly smartphone use, bedtime smartphone use, and smartphone use after waking) and prenatal affective disorders (PHQ-9 and GAD-7 total scores), Pearson correlation analysis was performed, with the results presented in Figure 1. The correlation matrix showed that both the SAS-SV total score and all dimensions of smartphone use duration were significantly positively correlated with PHQ-9 and GAD-7 total scores (*P*<.05), providing preliminary evidence that excessive digital media exposure was related to prenatal affective disorders.

**Figure 1.**
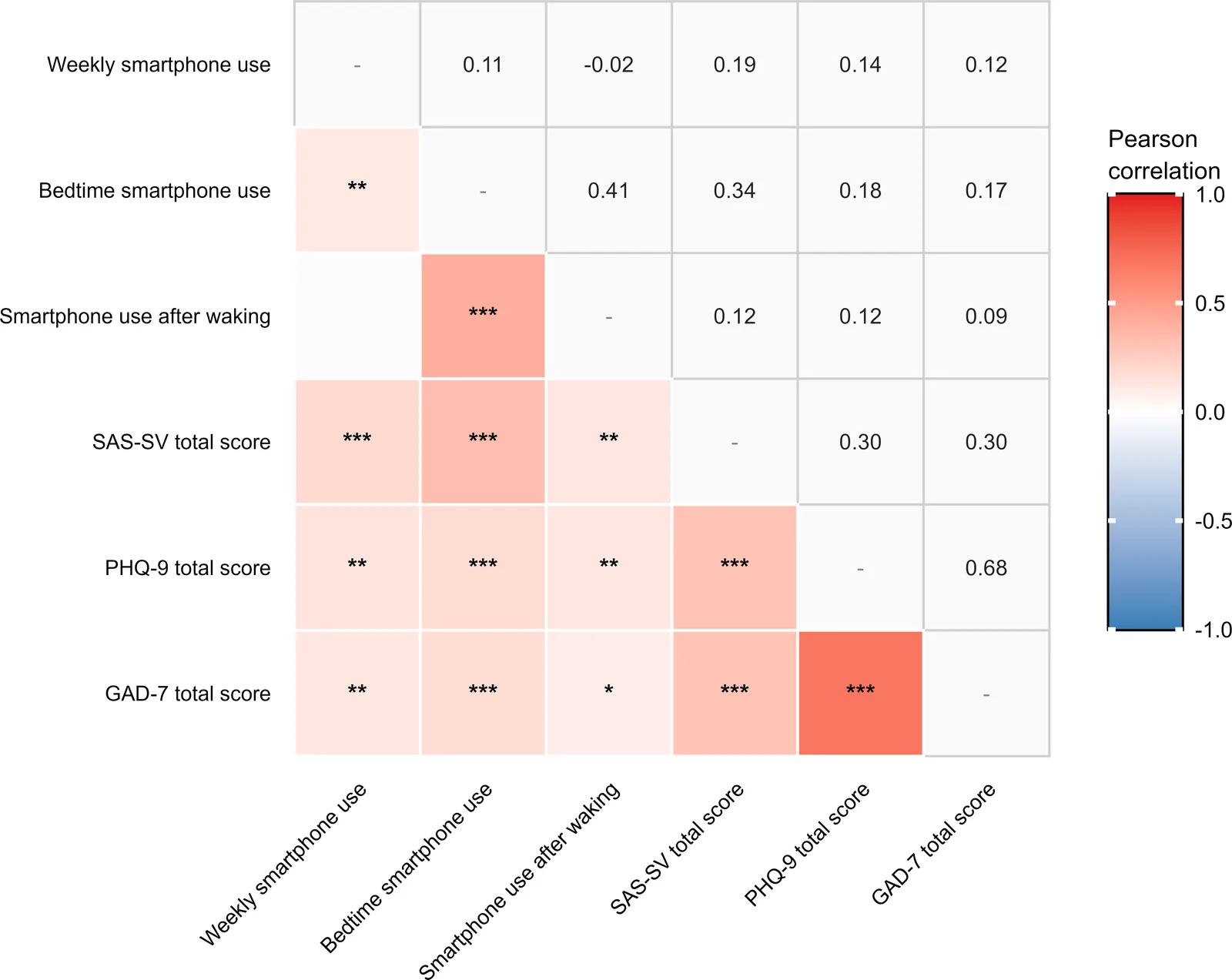
Correlation between different dimensions of smartphone use and prenatal affective disorders. These values are Pearson correlation coefficients (*r*). The dash “-” on the diagonal indicates that self-correlations were not computed (not applicable). *: *P* value <.05; **: *P* value <.01; ***: *P* value <.001. GAD-7: Generalized Anxiety Disorder-7; PHQ-9: Patient Health Questionnaire-9; SAS-SV: Smartphone Addiction Scale-Short Version.

### The Associations of Different Smartphone Use Dimensions and Sleep Quality With Prenatal Affective Disorders

The results of the independent associations between various dimensions of smartphone use and prenatal affective disorders are detailed in Table 2. Smartphone addiction was associated with higher odds of both outcomes in the crude and adjusted models. In the fully adjusted model, the OR was 3.461 (95% CI 1.771‐7.100) for prenatal depression and 7.738 (95% CI 3.064‐23.172) for prenatal anxiety. For bedtime use, compared with 0‐30 minutes, use of ≥91 minutes was associated with higher odds of depression (OR 3.716, 95% CI 1.467‐10.377) and anxiety (OR 5.141, 95% CI 1.568‐20.808). For use after waking, ≥61 minutes was associated with higher odds of depression (OR 4.758, 95% CI 1.736‐13.773) and anxiety (OR 10.902, 95% CI 2.606‐58.482). Weekly use showed a nonmonotonic categorical pattern: compared with 0‐14 hours/week, the 29-49 hours/week group had lower odds of depression (OR 0.378, 95% CI 0.119‐1.012) and anxiety (OR 0.221, 95% CI 0.033‐0.868), whereas the ≥50 hours/week group demonstrated elevated odds estimates for both outcomes (depression OR 1.687, 95% CI 0.795‐3.597; anxiety OR 1.979, 95% CI 0.830‐4.795). Sensitivity analyses using Firth penalized maximum likelihood estimation and an alternative threshold (PHQ-9 and GAD-7 score ≥5) yielded results consistent with the main findings in terms of direction, supporting the robustness of our results (Tables S1 and S2 in Multimedia Appendix 1).

RCS analyses showed that bedtime smartphone use and SAS-SV score had monotonic positive associations with both outcomes, without statistically significant evidence of nonlinearity. Smartphone use after waking showed a U-shaped association with depression (*P* for nonlinearity=.01) and a J-shaped association with anxiety (*P* for nonlinearity=.009). Weekly smartphone use showed a U-shaped association with anxiety (*P* for nonlinearity=.01), whereas its nonlinear association with depression was not statistically significant (Figures 2 and 3).

**Table 2.** Association between different dimensions of smartphone use and prenatal affective disorders^a^.

Exposure	Prenatal depression (PHQ-9^b^ score≥10)			Prenatal anxiety (GAD-7^c^ score≥10)		
n/N	Model 1^d^, OR^e^ (95% CI)	Model 2^f^, OR (95% CI)	n/N	Model 1^d^, OR (95% CI)	Model 2^f^, OR (95% CI)
SAS-SV^g^ total score (points)	—^h^	1.069 (1.035‐1.105)	1.070 **(**1.033‐1.110)	—	1.091 (1.049‐1.137)	1.104 (1.056‐1.158)
No	14/309	Ref^i^	Ref	6/309	Ref	Ref
Yes	33/228	3.566 (1.897‐7.041)	3.461 (1.771‐7.100)	25/228	6.219 (2.671‐16.984)	7.738 (3.064‐23.172)
Weekly smartphone use (hours)	—	1.099 (0.951‐1.256)	1.120 (0.959‐1.295)	—	1.139 (0.961‐1.327)	1.184 (0.990‐1.398)
0‐14	18/189	Ref	Ref	13/189	Ref	Ref
15‐28	6/87	0.704 (0.247‐1.749)	0.711 (0.238‐1.880)	2/87	0.319 (0.049‐1.187)	0.362 (0.053‐1.471)
29‐49	5/133	0.371 (0.120‐0.958)	0.378 (0.119‐1.012)	2/133	0.207 (0.032‐0.765)	0.221 (0.033‐0.868)
50+	18/128	1.555 (0.772‐3.133)	1.687 (0.795‐3.597)	14/128	1.663 (0.751‐3.708)	1.979 (0.830‐4.795)
Smartphone use after waking (minutes)	—	1.143 (1.004‐1.296)	1.171 **(**1.000**‐**1.349)	—	1.123 (0.956‐1.279)	1.207 (0.991‐1.433)
0‐1	8/152	Ref	Ref	3/152	Ref	Ref
2‐10	9/133	1.306 (0.485‐3.578)	1.399 (0.498‐4.011)	7/133	2.759 (0.750‐13.006)	3.375 (0.840‐17.270)
11‐60	16/187	1.694 (0.723‐4.284)	1.637 (0.667‐4.303)	13/187	3.732 (1.176‐16.503)	4.833 **(**1.375**‐**23.253)
61+	14/65	4.941 (1.998‐13.025)	4.758 **(**1.736**‐**13.773)	8/65	6.971 (1.941‐32.674)	10.902 **(**2.606**‐**58.482)
Bedtime smartphone use (minutes)	—	1.248 (1.079‐1.437)	1.238 **(**1.046**‐**1.461)	—	1.294 (1.092‐1.520)	1.361 **(**1.109**‐**1.666)
0‐30	7/170	Ref	Ref	4/170	Ref	Ref
31‐60	13/148	2.242 (0.892‐6.114)	1.964 (0.746‐5.548)	7/148	2.060 (0.609‐7.999)	2.386 (0.665‐9.938)
61‐90	8/84	2.451 (0.850‐7.228)	2.073 (0.685‐6.380)	7/84	3.773 (1.106‐14.760)	4.116 **(**1.115**‐**17.548)
91+	19/135	3.814 (1.618‐10.035)	3.716 **(**1.467**‐**10.377)	13/135	4.422 (1.523‐15.999)	5.141 **(**1.568**‐**20.808)

a Continuous exposure variables were SAS-SV total score, weekly smartphone use, smartphone use after waking, and bedtime smartphone use. For continuous exposures, ORs represent the change in odds per 1-point increase in SAS-SV total score, per 14-hour increase in weekly smartphone use, and per 30-minute increase in smartphone use after waking and bedtime smartphone use. A total SAS-SV score of 33 or higher is defined as smartphone addiction. Categorical smartphone use variables used the first category as the reference (Ref).

b PHQ-9: Patient Health Questionnaire-9.

c GAD-7: Generalized Anxiety Disorder-7.

d Model 1: crude model.

e OR: odds ratio.

f Model 2: adjustment for maternal age, maternal educational attainment, paternal educational attainment, prepregnancy smoking status, prepregnancy alcohol consumption, frequency of physical exercise during pregnancy, frequency of social media posts on platforms, prepregnancy BMI, weight gain during pregnancy, gravida, abortus, gestational age, and gestational diabetes mellitus.

g SAS-SV: Smartphone Addiction Scale-Short Version.

h Not available.

i Ref: reference.

**Figure 2.**
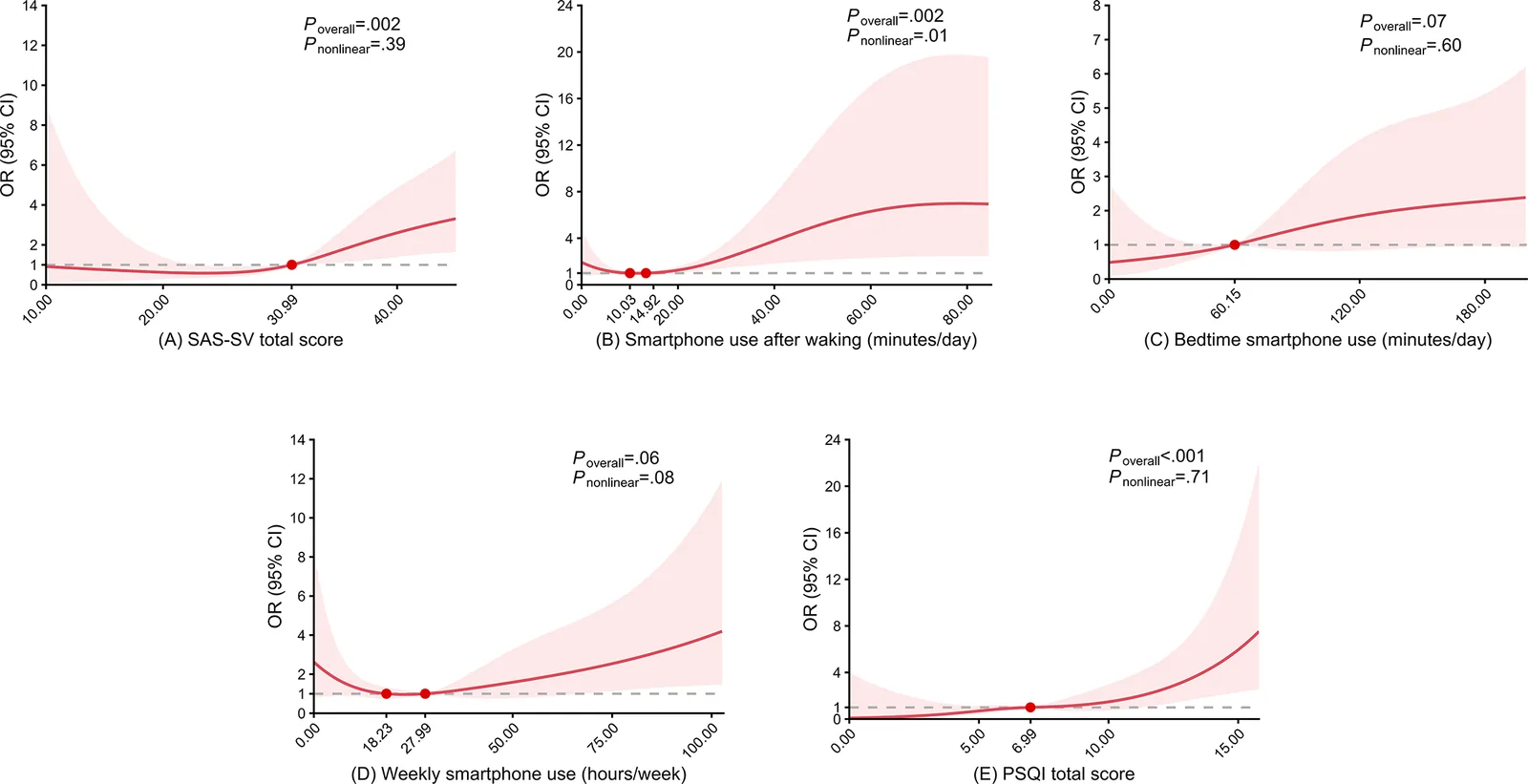
The dose-response relationships of (A) Smartphone Addiction Scale-Short Version total score, (B) smartphone use after waking, (C) bedtime smartphone use, (D) weekly smartphone use, and (E) Pittsburgh Sleep Quality Index total score with prenatal depression. The analyses in Figures (A) to (D) were adjusted for maternal age, maternal educational attainment, paternal educational attainment, pre-pregnancy smoking status, prepregnancy alcohol consumption, frequency of physical exercise during pregnancy, frequency of social media posts on platforms, prepregnancy body mass index, weight gain during pregnancy, gravida, abortus, gestational age, and gestational diabetes mellitus. The analyses in Figure (E) were adjusted for maternal age, maternal educational attainment, paternal educational attainment, pre-pregnancy smoking status, pre-pregnancy alcohol consumption, frequency of physical exercise during pregnancy, frequency of social media posts on platforms, pre-pregnancy body mass index, weight gain during pregnancy, gravida, abortus, gestational age, gestational diabetes mellitus, and weekly smartphone use. OR: odds ratio; PSQI: Pittsburgh Sleep Quality Index; SAS-SV: Smartphone Addiction Scale-Short Version.

**Figure 3.**
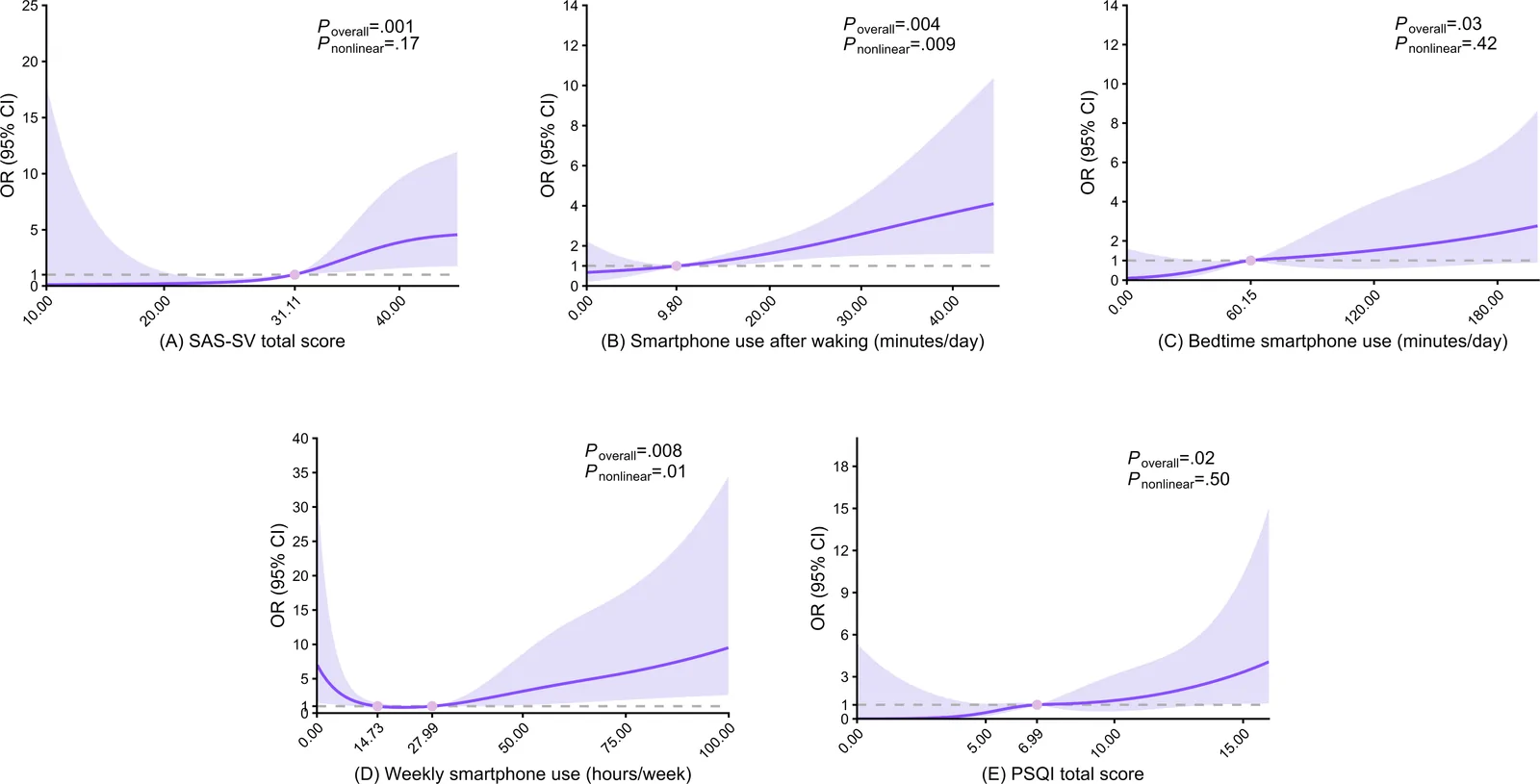
The dose-response relationships of (A) Smartphone Addiction Scale-Short Version total score, (B) smartphone use after waking, (C) bedtime smartphone use, (D) weekly smartphone use, and (E) Pittsburgh Sleep Quality Index total score with prenatal anxiety. The analyses in Figures (A) to (D) were adjusted for maternal age, maternal educational attainment, paternal educational attainment, pre-pregnancy smoking status, pre-pregnancy alcohol consumption, frequency of physical exercise during pregnancy, frequency of social media posts on platforms, pre-pregnancy body mass index, weight gain during pregnancy, gravida, abortus, gestational age, and gestational diabetes mellitus. The analyses in Figure (E) were adjusted for maternal age, maternal educational attainment, paternal educational attainment, pre-pregnancy smoking status, pre-pregnancy alcohol consumption, frequency of physical exercise during pregnancy, frequency of social media posts on platforms, pre-pregnancy body mass index, weight gain during pregnancy, gravida, abortus, gestational age, gestational diabetes mellitus, and weekly smartphone use. OR: odds ratio; PSQI: Pittsburgh Sleep Quality Index; SAS-SV: Smartphone Addiction Scale-Short Version.

Furthermore, the independent association between sleep quality and prenatal affective disorders was evaluated, with the results shown in Table 3. In the fully adjusted model, when the PSQI score was analyzed as a continuous variable, each 1-point increase in the score was associated with significantly higher odds of prenatal depression (OR 1.243, 95% CI 1.130‐1.373) and anxiety (OR 1.220, 95% CI 1.087‐1.374). The analysis using a categorical variable yielded consistent results: pregnant women with sleep disturbance (PSQI ≥7) had 2.732 times the odds of depression (OR 2.732, 95% CI 1.389‐5.592) and 2.702 times the odds of anxiety (OR 2.702, 95% CI 1.174‐6.657) compared with those with normal sleep. RCS analyses revealed that the PSQI score was positively associated with both prenatal depression and anxiety, although the nonlinear trends did not reach statistical significance (Figure 2E and Figure 3E).

**Table 3.** Association between sleep quality and prenatal affective disorders^a^.

Exposure	Prenatal depression (PHQ-9^b^ score≥10)		Prenatal anxiety (GAD-7^c^ score≥10)	
	Model 1^d^, OR^e^ (95% CI)	Model 2^f^, OR (95% CI)	Model 1^d^, OR (95% CI)	Model 2^f^, OR (95% CI)
PSQI^g^ total score	1.247 (1.142‐1.367)	1.243 (1.130‐1.373)	1.237 (1.115‐1.377)	1.220 (1.087‐1.374)
Poor sleep quality				
No	Ref	Ref	Ref	Ref
Yes	2.706 (1.453‐5.259)	2.732 (1.389‐5.592)	3.029 (1.410‐7.055)	2.702 **(**1.174‐6.657)

a For the continuous exposure variable (PSQI total score), the effect size reflects the change in the odds associated with a 1-point increase in the PSQI total score. A total PSQI score of 7 or higher is defined as indicating poor sleep quality.

b PHQ-9: Patient Health Questionnaire-9.

c GAD-7: Generalized Anxiety Disorder-7.

d Model 1: crude model.

e OR: odds ratio.

f Model 2: adjustment for maternal age, maternal educational attainment, paternal educational attainment, prepregnancy smoking status, prepregnancy alcohol consumption, frequency of physical exercise during pregnancy, frequency of social media posts on platforms, prepregnancy BMI, weight gain during pregnancy, gravida, abortus, gestational age, gestational diabetes mellitus, and weekly smartphone use.

g PSQI: Pittsburgh Sleep Quality Index.

### Mediating Effect Analysis

In this study, sleep quality was examined as a potential mediator in the associations between multidimensional smartphone use and prenatal affective disorders (Figures 4 and 5). Exploratory mediation analyses showed that the indirect pathways via sleep quality were statistically significant for the associations of bedtime smartphone use, weekly smartphone use, and smartphone addiction with prenatal depression, with mediation proportions of 44.89% (0.0065/0.0145; *P*<.001; Figure 4C), 31.30% (0.0056/0.0183; *P*=.04; Figure 4D), and 31.48% (0.0415/0.1315; *P*<.001; Figure 4A), respectively. Similar indirect patterns were observed in the anxiety models, with corresponding mediation proportions of 35.83% (0.0044/0.0123; *P*<.001; Figure 5C), 27.05% (0.0039/0.0144; *P*=.04; Figure 5D), and 22.73% (0.0271/0.1187; *P*<.001; Figure 5 B1). However, the indirect pathways from smartphone use after waking to depression and anxiety through sleep quality did not reach statistical significance (*P*=.12; Figure 4B and Figure 5B). Sensitivity mediation analyses using binary outcomes (PHQ-9 or GAD-7≥10) showed that the direction of indirect effects across pathways remained consistent with the main analysis, supporting the robustness of our mediation findings (Figures S1 and S2 in Multimedia Appendix 1).

**Figure 4.**
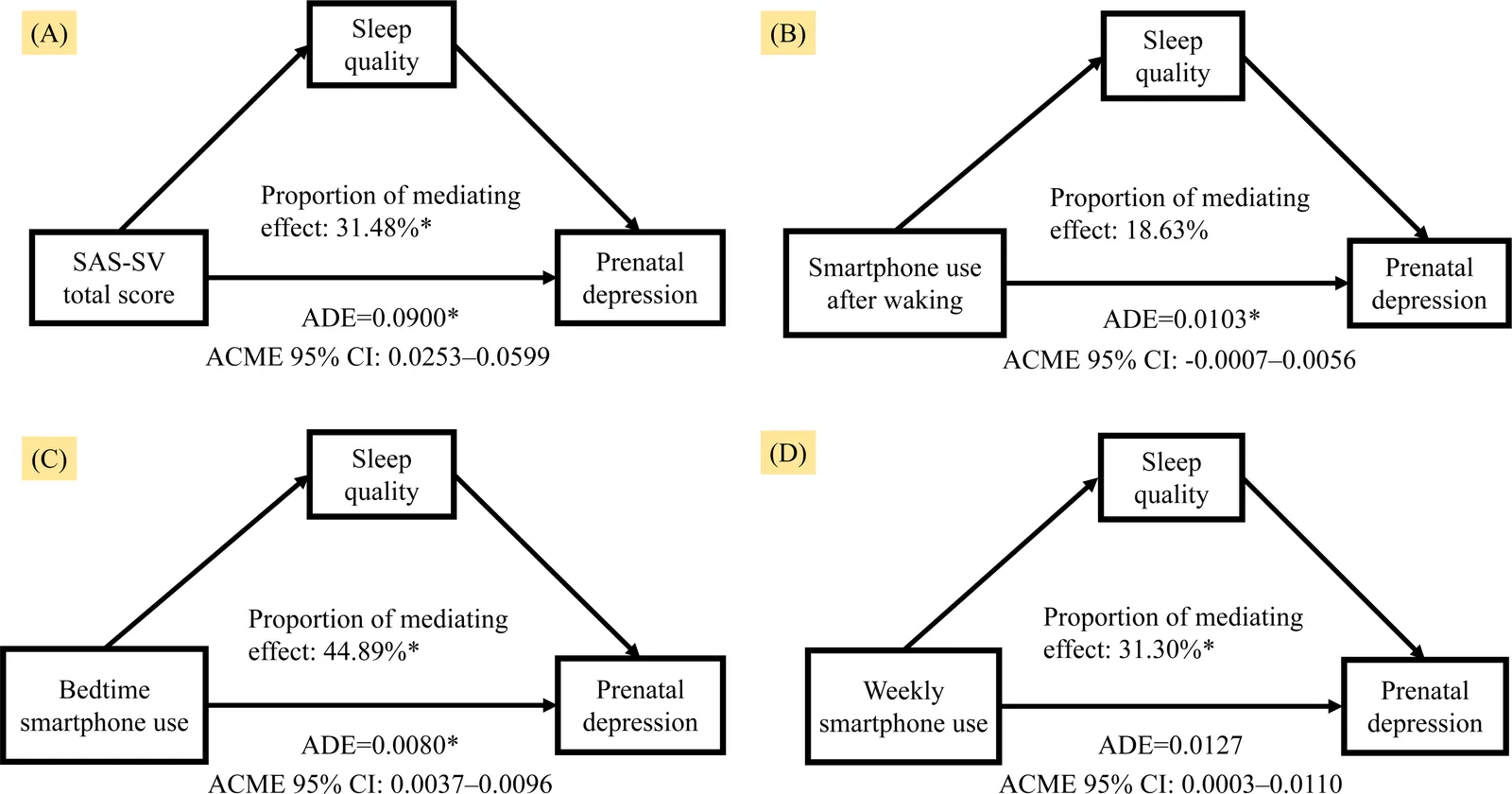
The mediation effect of sleep quality on the association between multidimensional smartphone use (Smartphone Addiction Scale-Short Version total score, smartphone use after waking, bedtime smartphone use, and weekly smartphone use) and prenatal depression using continuous outcomes (Patient Health Questionnaire-9 scores). Models were adjusted for maternal age, maternal educational attainment, paternal educational attainment, prepregnancy smoking status, pre-pregnancy alcohol consumption, frequency of physical exercise during pregnancy, frequency of social media posts on platforms, pre-pregnancy body mass index, weight gain during pregnancy, gravida, abortus, gestational age, and gestational diabetes mellitus. *: *P* value <.05. ACME: average causal mediation effect; ADE: average direct effect; SAS-SV: Smartphone Addiction Scale-Short Version.

**Figure 5.**
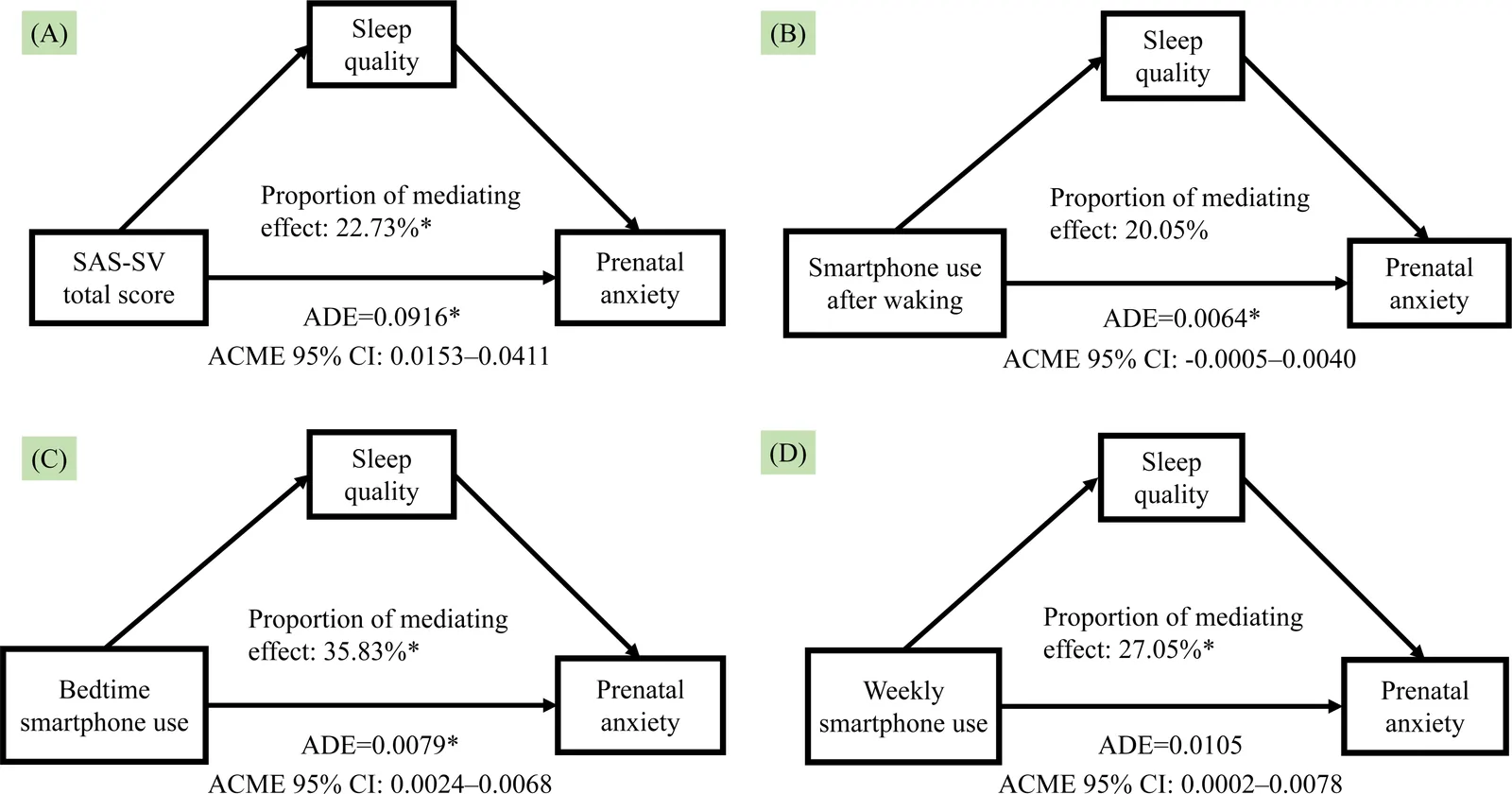
The mediation effect of sleep quality on the association between multidimensional smartphone use (Smartphone Addiction Scale-Short Version total score, smartphone use after waking, bedtime smartphone use, and weekly smartphone use) and prenatal anxiety using continuous outcomes (Generalized Anxiety Disorder-7 scores). Models were adjusted for maternal age, maternal educational attainment, paternal educational attainment, prepregnancy smoking status, prepregnancy alcohol consumption, frequency of physical exercise during pregnancy, frequency of social media posts on platforms, prepregnancy body mass index, weight gain during pregnancy, gravida, abortus, gestational age, and gestational diabetes mellitus. *: *P* value <.05. ACME: average causal mediation effect; ADE: average direct effect; SAS-SV: Smartphone Addiction Scale-Short Version.

## Discussion

### Principal Findings

This study systematically explored the associations of multidimensional smartphone use characteristics with prenatal depression and anxiety, and quantitatively validated the mediating role of sleep quality in these associations. The results showed that smartphone addiction and excessive use after waking and before bedtime were significantly associated with higher odds of prenatal affective disorders, and these associations remained robust after adjustment for multidimensional covariates. RCS analyses further revealed distinct dose–response relationships across different exposure dimensions: smartphone use after waking showed U-shaped and J-shaped associations with prenatal depression and anxiety, respectively; bedtime smartphone use and SAS-SV score showed monotonic positive associations. In addition, sleep quality partially mediated the associations of bedtime use, total weekly use, and smartphone addiction with prenatal depression and anxiety, with the mediation proportions varying across models.

The observed positive associations between smartphone addiction and prenatal depression and anxiety are consistent with findings from adolescents and college students [60-62]. In a meta-analysis encompassing 17 studies and 24,019 college students, Tang et al [63] reported that the pooled OR for the association between problematic smartphone use and depressive symptoms was 2.40 (95% CI 2.19‐2.63), which is lower than the estimate found in this study among pregnant women. This discrepancy suggests that pregnant women may be more susceptible to the adverse mental health effects of smartphone addiction. The potential mechanisms may involve 2 levels. From a neuroendocrine perspective, the dramatic fluctuations in pregnancy-related hormones such as estradiol, progesterone, and cortisol have already rendered the hypothalamic-pituitary-adrenal axis and the emotional regulation circuits of the limbic system in a fragile state of equilibrium; the withdrawal symptoms accompanying smartphone addiction may constitute an additional stress load that further disrupts an emotional homeostasis already operating at a compensatory margin [64,65]. From a sociobehavioral perspective, individuals with addiction tendencies tend to allocate a large amount of time to virtual social environments, thereby encroaching on the time budget that could otherwise be devoted to seeking family emotional support, offline health care visits, and self-care behaviors in the real world, consequently exacerbating the sense of social isolation that is characteristic of the prenatal period [66].

Notably, the associations of time-specific exposure demonstrated marked heterogeneity in this study. In the logistic regression analyses, bedtime smartphone use exceeding 91 minutes and smartphone use after waking exceeding 61 minutes were both significantly associated with higher odds of prenatal depression and anxiety. Previous studies have confirmed that bedtime and nocturnal smartphone use are associated with increased blue light exposure, which in turn has been linked to suppression of melatonin secretion and circadian phase delay [67-69]. However, the underlying mechanisms of digital exposure during the period after waking remain unclear. One possible explanation is that exposure to information on smartphones immediately after waking, such as work notifications and social media updates, may be associated with additional morning psychosocial stressors, which could in turn be related to fluctuations in daytime emotional states. Previous literature has suggested an association between smartphone use and alterations in neuroendocrine function, such as the cortisol awakening response [70]; however, as this study did not directly measure cortisol or other biomarkers, such pathways remain to be verified in future research. Therefore, the mechanisms linking smartphone use after waking to prenatal affective disorders should be interpreted with caution.

In this study, pregnant women with either very low or very high levels of smartphone use exhibited relatively higher odds of depression and anxiety, whereas those with a weekly use duration of 29‐49 hours had relatively lower effect estimates. This nonlinear pattern is similar to the U-shaped trend previously observed in the association between social media use and mental health [20]. From a clinical perspective, the use duration within this range may reflect pregnant women’s active needs for accessing pregnancy-related health information and maintaining social connections, rather than pathological overreliance [10]. Previous studies have suggested that daily smartphone use exceeding 4 hours is significantly associated with prenatal anxiety [12], which appears to contrast with the findings of this study. This discrepancy may indicate that the relationship between smartphone duration and mental health is moderated by multiple factors such as purpose of use, content, and individual susceptibility, rather than being a simple linear accumulation. In addition, the wide CIs for this point estimate and the inability to completely rule out residual confounding warrant a cautious interpretation of the findings.

The RCS analyses further revealed differences in nonlinear patterns across exposure dimensions. The dose-response relationship for duration of smartphone use after waking could not be adequately described by a simple monotonic linear function, with curves showing J-shaped and U-shaped patterns for anxiety and depression, respectively. The association between weekly total use duration and anxiety exhibited a U-shaped curve, whereas its nonlinear trend with depression was not statistically significant, suggesting that the between-group differences observed in Table 2 may partly arise from fluctuations introduced by the categorization strategy. Both duration of bedtime smartphone use and smartphone addiction score showed monotonically increasing associations with depression and anxiety, suggesting that higher exposure levels or greater addiction severity may be associated with higher odds of these outcomes. Thus, the adverse mental health correlates of smartphone use are not solely dependent on cumulative screen time but exhibit notable time-specific differences. Compared with focusing only on total weekly use duration, precise identification of and intervention on screen exposure during critical circadian windows may hold greater clinical value.

Exploratory mediation analyses further indicated that sleep quality partially accounted for the associations between multidimensional smartphone use and prenatal affective disorders, which is consistent with the mechanisms previously observed in nonpregnant populations [20,26,27]. Previous studies have demonstrated that excessive use of electronic devices such as smartphones is associated with increased blue light exposure, which in turn has been shown to suppress the normal secretory rhythm of pineal melatonin, prolong sleep onset latency, and delay circadian phase [67-69]. In addition, research has suggested that cognitive and emotional arousal triggered by immersive screen exposure before bedtime may interfere with sleep initiation and maintenance [29]. For pregnant women who already experience physiological sleep fragmentation, these effects may be further amplified. Previous research has also indicated that sleep deprivation is associated with impaired prefrontal cortex function and increased amygdala reactivity, which may weaken prefrontal-amygdala connectivity and contribute to emotion dysregulation [34,71]; insufficient sleep may also be associated with higher odds of depression and anxiety through activation of the sympathetic nervous system and inflammatory signaling pathways [72,73]. In this study, the mediation proportions of sleep quality in the associations of bedtime use, weekly use, and smartphone addiction with prenatal depression were 44.89% (0.0065/0.0145), 31.30% (0.0056/0.0183), and 31.48% (0.0415/0.1315), respectively; for prenatal anxiety, the corresponding proportions were 35.83% (0.0044/0.0123), 27.05% (0.0039/0.0144), and 22.73% (0.0271/0.1187). These findings suggest that sleep quality may represent a relevant pathway in these associations. Notably, the indirect pathways through sleep quality for smartphone use after waking were not statistically significant for either depression or anxiety. The specificity of these patterns suggests that the associations between digital device use at different times of day and prenatal mental health may operate through distinct biobehavioral pathways.

The association between prolonged and time-specific smartphone use and higher odds of prenatal affective disorders underscores the urgency of digital health management during pregnancy as an important public health issue. In this study, RCS analyses revealed a nonlinear association between smartphone use after waking and prenatal affective disorders, and identified a U-shaped pattern in the relationship between weekly smartphone use and affective disorders. This finding is consistent with the U-shaped association between social media use and mental health observed by Singh et al [74] in adolescent populations, suggesting that moderate smartphone use may be associated with lower emotional distress, whereas excessive use may be related to poorer mental health. These findings provide preliminary evidence for the development of prenatal digital health guidelines. These findings suggest that prenatal mental health interventions should not simply advocate a blanket reduction in smartphone use, but should instead shift toward more precise risk-stratification strategies, such as prioritizing screening for smartphone addiction tendencies and targeting screen exposure behaviors during high-risk periods, specifically after waking and before bedtime. Furthermore, the mediation proportion of sleep quality suggests that improving sleep may be a key intervention target for interrupting the pathway linking digital exposure to affective disorders. Cognitive behavioral interventions targeting bedtime smartphone use behavior may achieve the dual benefits of improving sleep and stabilizing mood simultaneously.

This study has several limitations. First, the study was conducted at the Hunan Provincial Maternal and Child Health Care Hospital, and the study population was limited to permanent residents who completed their entire prenatal care at that hospital; nearly 90 percent (475/537) of them held a college degree or higher, which may have influenced smartphone usage patterns: pregnant women with higher educational attainment may be more inclined to use smartphones to access prenatal health information, but they may also face a higher cognitive load from work-related information processing. Their smartphone usage behavior may reflect a dual characteristic of both health promotion and overreliance, which in turn limits the generalizability of this study’s conclusions to pregnant women with lower educational levels or those in rural areas. Second, the cross-sectional design limits our ability to infer causality; although the pathways in the mediation analysis were established based on evidence from previous longitudinal studies and biological plausibility, we cannot rule out the possibility of reverse pathways. Pregnant women may increase their smartphone use, particularly during periods of nighttime wakefulness, to pass the time or alleviate distress due to preexisting poor sleep quality or symptoms of depression and anxiety. This reverse causality may violate the temporal sequence assumption of the mediation model and could introduce bias into the estimates of indirect effects.

Furthermore, assessments of smartphone usage duration during specific time periods, smartphone addiction, and sleep quality relied on self-report questionnaires, which may introduce recall bias. In this study, total weekly usage duration was objectively recorded using the screen time feature built into the smartphone operating system; however, for usage durations after waking and before bedtime, self-reporting was used as an alternative because the existing operating system could not export data for specific time periods separately. This inconsistency in measurement methods may have some impact on the comparability of the results. Additionally, although we adjusted for several key covariates in our models, some theoretically important confounders (such as caffeine intake, dietary habits, sleep patterns, and occupation type) were not included in the analysis; their omission may lead to residual confounding, which could affect the precision of the effect estimates. Although this study included gestational diabetes mellitus as a covariate to control for the potential impact of common pregnancy complications, other unmeasured complications and lifestyle factors may still constitute residual confounding. However, given that the primary objective of this study was to explore the direction of the association and dose-response trends between smartphone usage patterns and prenatal affective disorders, the impact of these omissions on the directionality and statistical significance of the core conclusions is likely limited; nevertheless, more comprehensive covariate control would help yield more accurate effect estimates. Given the limited sample size in the anxiety group, the CIs for some estimates are relatively wide, indicating limited precision; therefore, the results should be interpreted with caution. Future large-scale, multicenter, prospective longitudinal cohort studies are needed to integrate objective data on screen time tracking and sleep monitoring, comprehensively collect the aforementioned covariates, and explore mediating mechanisms within a longitudinal research framework to further validate the findings of this study.

### Conclusion

The results of this study indicate that smartphone addiction and excessive smartphone use after waking and before bedtime were significantly associated with elevated odds of prenatal depression and anxiety, and distinct dose-response patterns exist across different exposure-outcome combinations. Additionally, exploratory mediation analyses indicated that sleep quality may statistically account for part of these associations. Future longitudinal studies are needed to establish temporal precedence and to test the directional assumptions underlying the mediation model.

## Supplementary material

10.2196/100064 Multimedia Appendix 1 Results of sensitivity mediation analyses.

## Abbreviations

GAD-7: Generalized Anxiety Disorder-7
OR: odds ratio
PHQ-9: Patient Health Questionnaire-9
PSQI: Pittsburgh Sleep Quality Index
RCS: restricted cubic spline
SAS-SV: Smartphone Addiction Scale-Short Version
