# Supplementary material for: The Mediating Role of Sleep Quality in Associations of Smartphone Addiction and Time-Specific Use With Prenatal Depression and Anxiety: Cross-Sectional Observational Study
Source: JMIR Mhealth Uhealth. 2026 Oct 30;14:e100064. doi: 10.2196/100064 (PMC13721738; doi:10.2196/100064)
Supplement: Supplementary file 1 [file mhealth-v14-e100064-s001.docx]

**Table S1. Association between different dimensions of smartphone use and prenatal affective disorders using Firth's penalized maximum likelihood estimation.**

| **Exposure** | **Prenatal Depression (PHQ-9 score ≥10)** | | **Prenatal Anxiety (GAD-7 score ≥10)** | |
| --- | --- | --- | --- | --- |
|  | **Model1 [OR (95%CI)]** | **Model2 [OR (95%CI)]** | **Model1 [OR (95%CI)]** | **Model2 [OR (95%CI)]** |
| SAS-SV total score (point) | 1.068(1.034-1.104) | **1.054(1.018-1.093)** | 1.090(1.049-1.135) | **1.083(1.037-1.137)** |
| No | Ref | Ref | Ref | Ref |
| Yes | 3.492(1.872-6.826) | **2.994(1.547-6.141)** | 5.851(2.572-15.368) | **5.049(2.129-13.911)** |
| Weekly smartphone use (h) | 1.104(0.956-1.259) | 1.156(0.995-1.338) | 1.146(0.970-1.331) | **1.189(1.006-1.401)** |
| 0–14 | Ref | Ref | Ref | Ref |
| 15–28 | 0.739(0.270-1.795) | 0.848(0.292-2.235) | 0.382(0.074-1.298) | 0.570(0.113-2.054) |
| 29–49 | 0.397(0.135-0.994) | 0.591(0.202-1.533) | 0.249(0.048-0.838) | 0.396(0.077-1.395) |
| 50+ | 1.552(0.777-3.101) | 1.909(0.894-4.148) | 1.656(0.758-3.642) | 2.251(0.954-5.542) |
| Smartphone use after waking (min) | 1.144(1.017-1.287) | 1.104(0.951-1.270) | 1.132(0.984-1.275) | 1.159(0.952-1.430) |
| 0–1 | Ref | Ref | Ref | Ref |
| 2–10 | 1.297(0.495-3.452) | 1.303(0.497-3.479) | 2.532(0.736-10.606) | 3.294(0.896-14.580) |
| 11–60 | 1.645(0.715-4.057) | 1.310(0.557-3.275) | 3.324(1.109-13.063) | **3.562(1.098-14.769)** |
| 61+ | 4.786(1.973-12.305) | **2.822(1.026-8.045)** | 6.314(1.873-26.337) | **4.336(1.074-20.546)** |
| Bedtime smartphone use (min) | 1.250(1.082-1.436) | **1.193(1.003-1.412)** | 1.296(1.098-1.518) | **1.270(1.036-1.549)** |
| 0–30 | Ref | Ref | Ref | Ref |
| 31–60 | 2.172(0.884-5.740) | 1.551(0.630-4.060) | 1.961(0.611-7.051) | 1.646(0.524-5.765) |
| 61–90 | 2.422(0.868-6.898) | 1.368(0.453-4.040) | 3.581(1.106-12.979) | 2.189(0.643-7.989) |
| 91+ | 3.649(1.579-9.315) | **2.997(1.211-7.995)** | 4.078(1.463-13.701) | **3.181(1.051-11.099)** |

**Note**: OR, odds ratio; CI, confidence interval; SAS-SV, Smartphone Addiction Scale-Short Version.

Firth's penalized maximum likelihood estimation was applied to reduce small-sample bias in rare-events logistic regression.

The effect sizes for continuous exposure variables reflect the change in the odds associated with a 1-point increase in the SAS-SV total score, a 14-hour increase in weekly smartphone use, and a 30-minute increase in smartphone use after waking and bedtime smartphone use. A total SAS-SV score of 33 or higher is defined as smartphone addiction.

Model 1, crude model

Model 2, adjustment for maternal age, maternal educational attainment, paternal educational attainment, pre-pregnancy smoking status, pre-pregnancy alcohol consumption, frequency of physical exercise during pregnancy, frequency of social media posts on platforms, pre-pregnancy body mass index, weight gain during pregnancy, gravida, abortus, gestational age and gestational diabetes mellitus.

**Table S2. Association between different dimensions of smartphone use and prenatal affective disorders**

| **Exposure** | **Prenatal Depression (PHQ-9 score ≥5)** | | **Prenatal Anxiety (GAD-7 score ≥5)** | |
| --- | --- | --- | --- | --- |
|  | **Model1 [OR (95%CI)]** | **Model2 [OR (95%CI)]** | **Model1 [OR (95%CI)]** | **Model2 [OR (95%CI)]** |
| SAS-SV total score (point) | 1.066(1.044-1.089) | **1.066(1.042-1.092)** | 1.059(1.037-1.083) | **1.061(1.037-1.087)** |
| No | Ref | Ref | Ref | Ref |
| Yes | 2.110(1.483-3.011) | **2.121(1.447-3.121)** | 2.938(2.015-4.311) | **2.973(1.989-4.477)** |
| Weekly smartphone use (h) | 1.092(1.001-1.193) | 1.076(0.979-1.184) | 1.015(0.924-1.113) | 1.007(0.910-1.110) |
| 0–14 | Ref | Ref | Ref | Ref |
| 15–28 | 1.285(0.761-2.161) | 1.487(0.846-2.610) | 0.652(0.360-1.148) | 0.670(0.358-1.219) |
| 29–49 | 0.811(0.503-1.299) | 0.760(0.455-1.260) | 0.850(0.523-1.371) | 0.827(0.494-1.373) |
| 50+ | 1.879(1.191-2.975) | **1.866(1.142-3.061)** | 0.965(0.595-1.556) | 0.927(0.557-1.535) |
| Smartphone use after waking (min) | 1.128(1.013-1.279) | **1.215(1.072-1.401)** | 1.077(0.970-1.203) | 1.101(0.978-1.245) |
| 0–1 | Ref | Ref | Ref | Ref |
| 2–10 | 1.224(0.748-2.004) | 1.049(0.614-1.790) | 1.487(0.882-2.518) | 1.477(0.842-2.602) |
| 11–60 | 1.600(1.024-2.516) | 1.608(0.985-2.645) | 1.572(0.974-2.565) | 1.608(0.959-2.724) |
| 61+ | 2.376(1.314-4.330) | **3.022(1.523-6.088)** | 1.886(1.002-3.529) | **2.165(1.071-4.369)** |
| Bedtime smartphone use (min) | 1.206(1.093-1.335) | **1.237(1.106-1.386)** | 1.146(1.036-1.269) | **1.146(1.024-1.283)** |
| 0–30 | Ref | Ref | Ref | Ref |
| 31–60 | 1.845(1.149-2.982) | **2.055(1.239-3.436)** | 1.247(0.755-2.061) | 1.285(0.757-2.182) |
| 61–90 | 2.482(1.435-4.313) | **2.677(1.481-4.875)** | 1.936(1.100-3.404) | **2.009(1.098-3.678)** |
| 91+ | 2.906(1.804-4.729) | **3.340(1.964-5.749)** | 1.736(1.057-2.865) | 1.699(0.989-2.929) |

**Note**: OR, odds ratio; CI, confidence interval; SAS-SV, Smartphone Addiction Scale-Short Version.

The effect sizes for continuous exposure variables reflect the change in the odds associated with a 1-point increase in the SAS-SV total score, a 14-hour increase in weekly smartphone use, and a 30-minute increase in smartphone use after waking and bedtime smartphone use. A total SAS-SV score of 33 or higher is defined as smartphone addiction.

Model 1, crude model

Model 2, adjustment for maternal age, maternal educational attainment, paternal educational attainment, pre-pregnancy smoking status, pre-pregnancy alcohol consumption, frequency of physical exercise during pregnancy, frequency of social media posts on platforms, pre-pregnancy body mass index, weight gain during pregnancy, gravida, abortus, gestational age and gestational diabetes mellitus.

**Figure S1.** The mediation effect of sleep quality on the association between multidimensional smartphone use (SAS-SV total score, smartphone use after waking, bedtime smartphone use, and weekly smartphone use) and prenatal depression using binary outcomes (PHQ-9 ≥ 10). Models were adjusted for maternal age, maternal educational attainment, paternal educational attainment, pre-pregnancy smoking status, pre-pregnancy alcohol consumption, frequency of physical exercise during pregnancy, frequency of social media posts on platforms, pre-pregnancy body mass index, weight gain during pregnancy, gravida, abortus, gestational age, and gestational diabetes mellitus. ADE: average direct effect. ACME: average causal mediation effect.

**Figure S2.** The mediation effect of sleep quality on the association between multidimensional smartphone use (SAS-SV total score, smartphone use after waking, bedtime smartphone use, and weekly smartphone use) and prenatal anxiety using binary outcomes (GAD-7 ≥ 10). Models were adjusted for maternal age, maternal educational attainment, paternal educational attainment, pre-pregnancy smoking status, pre-pregnancy alcohol consumption, frequency of physical exercise during pregnancy, frequency of social media posts on platforms, pre-pregnancy body mass index, weight gain during pregnancy, gravida, abortus, gestational age, and gestational diabetes mellitus. ADE: average direct effect. ACME: average causal mediation effect.
